# Combined biologic therapy targeting B cells and eosinophils in relapsing multiple sclerosis and severe asthma: a case report of ofatumumab and mepolizumab

**DOI:** 10.3389/fimmu.2025.1634477

**Published:** 2025-12-18

**Authors:** Aurora Zanghì, Paola Sofia Di Filippo, Claudia Rutigliano, Carlo Avolio, Emanuele D’Amico

**Affiliations:** BRAND Center, Breakthrough Research in Autoimmune and Neurodegenerative Diseases, Department of Medical and Surgical Sciences, University of Foggia, Foggia, Italy

**Keywords:** multiple sclerosis, combination therapy, ofatumumab, mepolizumab, monoclonal antibodies, asthma

## Abstract

The management of multiple sclerosis (MS) in patients with significant comorbidities such as severe asthma presents unique therapeutic challenges. We report a case of successful combined biologic therapy with ofatumumab and mepolizumab in a patient with relapsing MS and pre-existing severe eosinophilic asthma, highlighting the feasibility and safety of dual biologic therapy. To our knowledge, this is the first report of concomitant use of ofatumumab and mepolizumab in a patient with MS and severe eosinophilic asthma, documenting the safety of a non-standard combination strategy in a real-world clinical scenario.

## Introduction

Multiple sclerosis (MS) is a chronic, immune-mediated disorder of the central nervous system (CNS) characterized by inflammation, demyelination, and neurodegeneration (1). The pathogenesis of MS is complex, with B lymphocytes playing a pivotal role in antigen presentation, cytokine secretion, and the formation of ectopic lymphoid follicles within the CNS (2).

Over recent years, the advent of B-cell depleting therapies has significantly impacted the therapeutic landscape of MS, offering improved disease control and reduced relapse rates (2, 3). Ofatumumab, a fully human anti-CD20 monoclonal antibody, has demonstrated robust efficacy in relapsing forms of MS by selectively targeting and depleting circulating B cells, thereby attenuating the autoimmune cascade responsible for demyelination and axonal injury (2).

The management of MS is further complicated in patients with significant comorbidities, particularly those with history of severe, treatment-refractory asthma.

In such cases, the interplay between chronic systemic inflammation and immune dysregulation may influence both disease progression and therapeutic response (4).

Here, we describe the clinical course and outcomes of a patient with relapsing-remitting MS and previously diagnosed severe asthma, treated with mepolizumab that started ofatumumab.

## Case presentation

Here we present the case of a 61-year-old male with a 19-year history of relapsing-remitting MS and severe eosinophilic asthma, regularly monitored at our center with a structured multidisciplinary follow-up.

His MS treatment history includes dimethyl fumarate from 2016 to 2017 and teriflunomide from 2019 until September 2024.

The patient’s follow-up neurological protocol consists of clinical assessments, blood tests (including lymphocytic count and immunophenotype characterization serum immunoglobulin levels, and virological screening for common latent viruses (Epstein–Barr virus, Cytomegalovirus, Hepatitis B virus, and Hepatitis C virus) every three months, brain MRI and cognitive monitoring through the Brief International Cognitive Assessment for MS (BICAMS) every six months (5).

In August 2024, the patient experienced a clinical relapse characterized by postural instability, with only partial recovery after intravenous corticosteroid pulse therapy. Neurological examination revealed an EDSS score of 4.0. Cognitive assessment showed a decline in episodic memory and lexical access, as documented by BICAMS. The baseline MRI revealed no new T2-weighted lesions or new Gadoliunium enhancing lesions.

Given the clinical and cognitive progression, teriflunomide was discontinued and ofatumumab therapy was initiated (20 mg subcutaneously once weekly for three weeks, then monthly) in October 2024. Prior to initiating ofatumumab, the patient had received standard and recommended immunizations in accordance with national preventive care recommendations. Baseline laboratory investigations showed normal IgG levels and a B-lymphocyte count within the expected range.

Ofatumumab was initiated despite the patient already being on long-term therapy with mepolizumab (100 mg subcutaneously every 4 weeks since 2020) for severe eosinophilic asthma, diagnosed in 2016. Mepolizumab had been prescribed according to the Italian Medicines Agency (AIFA) rules, after insufficient asthma control with maximal inhaled therapy and recurrent exacerbations requiring an average of 3–4 courses of oral corticosteroids per year.

Following mepolizumab initiation, the frequency of exacerbations progressively decreased, with only one corticosteroid-requiring episode in the year prior to ofatumumab initiation.

Pulmonary follow-up also included routine use of the Asthma Control Test (ACT) and spirometry, as performed by the treating pulmonologists to further support the patient’s stable respiratory condition. (6, 7).

After 12 months of combined biologic therapy, the patient remained neurologically stable, with an unchanged EDSS of 4.0 and a stable BICAMS evaluation. MRI revealed no new T2 or gadolinium-enhancing lesions. Laboratory monitoring showed sustained B-cell depletion (counts <10/μL) and stable IgG levels. Asthma control was maintained, with no exacerbations and no need for oral corticosteroids, while routine evaluations confirmed stability. No serious adverse events were observed during this period of dual biologic therapy (see **Table 1**).

**Table 1:** Clinical and laboratory parameters at baseline and 12-month follow-up.

Parameter	Baseline	12-months follow-up
EDSS	4.0	4.0
New lesions in T2 weighted MRI sequences	0*	0
New lesions in T1-Gd+ weighted MRI sequences	0*	0
B-lymphocytes (cells/μL)	180	<10
IgG (mg/dL)	710	698
FEV1, % predicted)	78	81
ACT score	18	23
Blood eosinophils (cells/μL)	0	0
Asthma exacerbations	1*	0
Serious adverse events	/*	None

EDSS, Expanded Disability Status Scale.

MRI, Magnetic Resonance Imaging.

T2 lesions, Hyperintense lesions on T2-weighted MRI sequences.

Gd+, Gadolinium-enhancing lesions.

B-lymphocytes, B cell count in peripheral blood (reference 90-660 cell per microliter).

IgG, Immunoglobulin G (mg/dL).

FEV1: Forced Expiratory Volume in 1 second, expressed as percentage of predicted value.

ACT, Asthma Control Test (score range: 5–25; higher scores indicate better asthma control).

Blood eosinophils: Eosinophil count in peripheral blood (cells per microliter).

Asthma exacerbations: Number of asthma exacerbations in the previous year.

Serious adverse events: Any serious adverse events reported during the observation period.

*in the previous 12 months.

## Discussion

This case describes a patient with MS and severe eosinophilic asthma treated with concurrent ofatumumab and mepolizumab. The patient maintained neurological stability and experienced sustained asthma control without exacerbations. Clinical and laboratory monitoring confirmed effective B-cell depletion, persistent eosinophil suppression, and stable immunoglobulin levels. No infectious complications or adverse events were observed.

Additionally, our findings align with a growing body of literature challenging the traditional binary model of MS activity based solely on clinical relapses or radiological changes. Cognitive decline, especially when acute and measurable, is increasingly recognized as a valid marker of disease activity, sometimes occurring in isolation or dissociated from MRI progression (8). Recent longitudinal studies have shown that even in neurologically stable patients, cognitive deterioration may represent an early sign of progression or subclinical inflammation (8, 9).

These insights support the concept of MS as a continuum, where radiological and clinical markers may diverge, and underline the importance of including neuropsychological metrics in routine disease activity assessment.

Currently, monotherapy is the only approved and standard approach for MS treatment. This paradigm, while established, may limit therapeutic options, particularly for patients with complex immune-mediated comorbidities (10, 11). In contrast, other medical specialties, such as rheumatology, routinely employ integrated, polytherapeutic strategies to target multiple pathogenic pathways and optimize patient outcomes (12).

**Figure 1:**
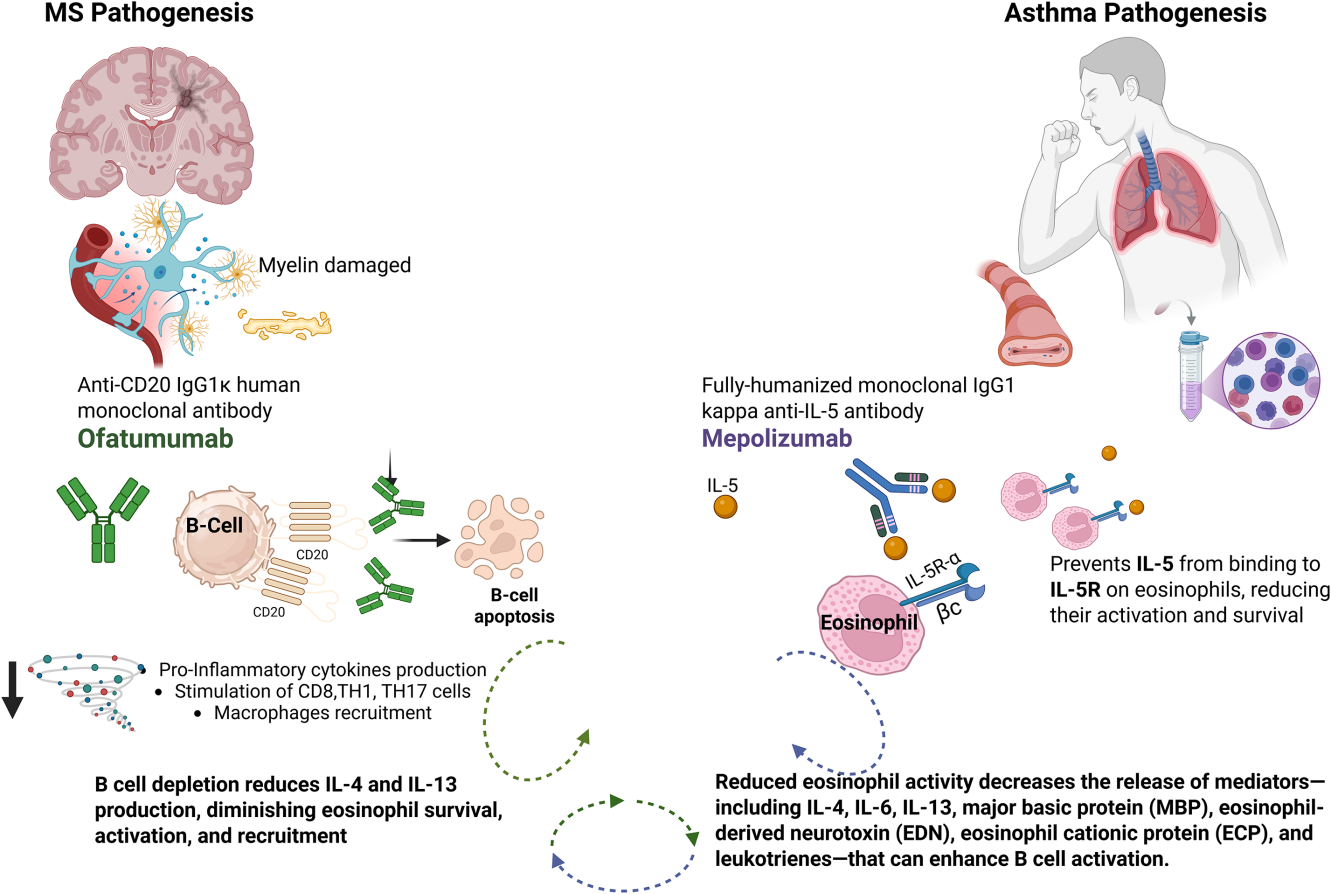
Ofatumumab and mepoluzimab mechanisms of action. Created with BioRender.com.

The immunological rationale for a combined approach in MS could be supported by the complementary mechanisms of action of different agents. B-cell depletion with ofatumumab may reduce pro-inflammatory cytokine production relevant to both MS and eosinophilic inflammation, while IL-5 inhibition with mepolizumab attenuates eosinophil-mediated tissue damage, which may also influence neuroinflammation (**Figure 1**). A growing body of evidence supports the notion that IL-5 plays a role not only in eosinophil biology but also in modulating B-cell function. Experimental models show that IL-5 promotes B-cell proliferation, survival, and differentiation—particularly of B cells—into antibody-secreting plasma cells (13). The IL-5 receptor (IL-5Rα) is expressed on subsets of activated B cells, and its stimulation can synergize with other pathways to promote immunoglobulin class-switch recombination and plasma cell differentiation. Moreover, eosinophil-derived mediators have been shown to enhance B-cell responses, further linking the eosinophilic axis to humoral immunity (14).

This mechanistic overlap reinforces the translational rationale for targeting both B cells and eosinophils in selected cases with overlapping immune-mediated conditions, as in our patient.

The observed safety profile in this case is consistent with established data for each agent, though vigilance for cumulative immunosuppression remains warranted, particularly regarding infection risk in the long term follow up.

The key contribution of this case is the documentation of safety and feasibility of combining two biologic therapies with distinct immunological targets in a patient with MS and a severe immune-mediated comorbidity. Although the use of dual biologic therapy in MS remains rare, its application is increasingly explored in other autoimmune and immune-mediated diseases. For instance, in rheumatoid arthritis and psoriatic arthritis, the combination of biologics targeting different inflammatory pathways (e.g., TNF inhibitors with IL-17 or IL-6 blockers) has shown clinical potential in refractory cases, despite limited safety data (15). Similarly, in inflammatory bowel diseases, dual biologic regimens have been employed in patients with high disease burden or secondary loss of response, with emerging reports of clinical and endoscopic improvement (16).

While data are still limited, these precedents suggest that, under careful monitoring, dual biologic strategies may be feasible in complex or overlapping immunologic conditions and our experience provides preliminary real-world data that may help inform neurologists facing similar complex scenarios.

This case also underlines the importance of a multidisciplinary approach in patients with MS and significant immune-mediated comorbidities. In our patient, continuation of mepolizumab was determined by the treating pulmonologist according to established national guidelines, while ofatumumab was introduced for MS disease reactivation. The coordinated management ensured that the patient did not have to discontinue an effective therapy for asthma when starting a high efficacy disease modifying therapy for MS.

Further studies are needed to establish the safety, efficacy, and selection criteria for combination therapy approaches in MS, with the goal of optimizing outcomes for both MS and associated comorbidities.

## Data Availability

The raw data supporting the conclusions of this article will be made available by the authors, without undue reservation.
